# Comparison of machine learning methods to predict udder health status based on somatic cell counts in dairy cows

**DOI:** 10.1038/s41598-021-93056-4

**Published:** 2021-07-01

**Authors:** Tania Bobbo, Stefano Biffani, Cristian Taccioli, Mauro Penasa, Martino Cassandro

**Affiliations:** 1 Department of Agronomy, Food, Natural resources, Animals and Environment (DAFNAE), University of Padova, Viale dell’Università 16, 35020 Legnaro, PD Italy; 2 Istituto Di Biologia E Biotecnologia Agraria, Consiglio Nazionale Delle Ricerche, Via Edoardo Bassini 15, 20133 Milano, Italy; 3 Department of Animal Medicine, Production and Health (MAPS), University of Padova, Viale dell’Università 16, 35020 Legnaro, PD Italy

**Keywords:** Machine learning, Animal breeding, Animal physiology

## Abstract

Bovine mastitis is one of the most important economic and health issues in dairy farms. Data collection during routine recording procedures and access to large datasets have shed the light on the possibility to use trained machine learning algorithms to predict the udder health status of cows. In this study, we compared eight different machine learning methods (Linear Discriminant Analysis, Generalized Linear Model with logit link function, Naïve Bayes, Classification and Regression Trees, k-Nearest Neighbors, Support Vector Machines, Random Forest and Neural Network) to predict udder health status of cows based on somatic cell counts. Prediction accuracies of all methods were above 75%. According to different metrics, Neural Network, Random Forest and linear methods had the best performance in predicting udder health classes at a given test-day (healthy or mastitic according to somatic cell count below or above a predefined threshold of 200,000 cells/mL) based on the cow’s milk traits recorded at previous test-day. Our findings suggest machine learning algorithms as a promising tool to improve decision making for farmers. Machine learning analysis would improve the surveillance methods and help farmers to identify in advance those cows that would possibly have high somatic cell count in the subsequent test-day.

## Introduction

Bovine mastitis, an inflammatory condition of the mammary gland most commonly caused by bacterial infection, is a significant health issue in dairy farms. It strongly affects cow welfare and longevity, leading to economic losses due to reduced milk production, poor milk quality and treatments cost^1,2^. Different strategies are nowadays available to achieve and maintain a good udder health status of dairy cows, including the improvement of the herd hygienic conditions, the genetic selection of animals to enhance resistance to mastitis, and the improvement of mastitis detection systems^3^. Bacteriological analysis and PCR assay are the best methods to identify intramammary infections in cows^4^. However, these methods cannot be applied to routine data collection at population level as they are expensive and time-consuming. Therefore, milk somatic cell count (SCC), which reflects the inflammatory status of the mammary gland, has been extensively used to monitor udder health and milk quality^5^ and to genetically reduce the susceptibility of cows to mastitis^6^. Nevertheless, the combined use of different indirect indicators of mastitis could be more successful to detect the disease. For this reason, novel indicators of mammary gland inflammation have been explored, including alternative traits describing SCC variation throughout the lactation^7–9^, cellular immune-associated traits^10^, proteins measured in blood serum^11,12^, and detailed analysis of the different cell types in milk, i.e., polymorphonuclear neutrophils, macrophages and lymphocytes, whose proportion varies in milk according to the inflammatory status of the udder^13^. Thanks to the recent implementation of novel milk-testing technologies based on flow cytometry in different laboratories of the Italian Breeders Associations (Rome, Italy), differential somatic cell count (DSCC), i.e., the ratio of neutrophils and lymphocytes to total SCC^13^, is monthly recorded with the aim of improving the identification of the mammary gland status of dairy cows. Indeed, the combined use of SCC and DSCC is more accurate to screen for mastitis than SCC only^14^. In fact, it allows the identification of (1) healthy cows with low SCC and DSCC, (2) susceptible cows with low SCC but high DSCC (i.e., those where an immune response has begun and an increase in neutrophils is present^15^), (3) mastitic cows with high SCC and DSCC, and (4) cows affected by chronic mastitis (those with high SCC but low DSCC, as macrophages are the predominant cell type^16^). Although a monthly mastitis risk ratio based on a combination of SCC and DSCC is currently provided to the farmers by the Italian Breeders Association, additional efforts are needed not only to manage mastitis in the farm but also to prevent it. Therefore, the development of new reliable methods to predict mastitis occurrence is a priority. Routine data collection during monthly recording procedures and access to large datasets including information on herd, cows and milk composition suggest the possibility to use machine learning (ML) classification algorithms to predict the udder health status of cows. The ML methods allow identification of meaningful relationships between variables and exploit this information to train different prediction models and evaluate their predictive performance on unknown data. Different studies have applied ML to predict and diagnose mastitis, defined by the presence of high milk SCC^17–19^ or mastitis pathogens^20–23^. Some of these studies^17–19^ have established SCC-independent models for mastitis prediction, testing different prediction models on milking traits (e.g., milk volume, fat, protein, lactose, electrical conductivity, milking time and peak flow) obtained by the use of automatic milking systems to predict subclinical mastitis (SCC ≥ 250,000 cells/mL). Nevertheless, in those studies both outcome (mastitic or healthy according to SCC value) and features (milk traits) were recorded at the same milking time. Predictions based on data collected at different time points were reported by Anglart et al.^24^, who explored different ML methods for predicting cow composite SCC by using quarter and cow milk data regularly recorded in cows milked with automatic milking system. However, data spanned a 2-mo period and were collected in only one dairy farm. Moreover, prediction of subclinical mastitis using information (i.e., animal information, milk production and composition) recorded on the previous test-day (TD) in the frame of the monthly recording procedure across several dairy herds have not been investigated yet. Such analysis would improve the surveillance methods and help farmers to identify in advance those cows that would possibly present high SCC level in the subsequent TD. Therefore, the aim of the present study was to compare the performance of ML algorithms in predicting the udder health status of cows (healthy or mastitic according to SCC below or above a predefined threshold) at TD *n* + *1* using cow and milk information collected at the previous TD *n*.

## Results

Prediction models developed within eight different ML methods, namely Linear Discriminant Analysis (LDA), Generalized Linear Model (GLM) with logit link function, Naïve Bayes (NB), Classification and Regression Trees (CART), k-Nearest Neighbors (kNN), Support Vector Machines (SVM), Random Forest (RF) and Neural Network (NN) were trained and tested on 80% of the data (14,755 records) to identify the best udder health prediction method based on data previously recorded on cow (parity and stage of lactation) and milk (year of sampling, season of sampling, milk yield, fat, protein, casein, lactose, pH, urea, log_10_SCC, DSCC, and average milk yield and log_10_SCC of cows of the same contemporary group, defined as cows sampled in the same herd and day).

### Recursive feature selection

**Figure 1:**
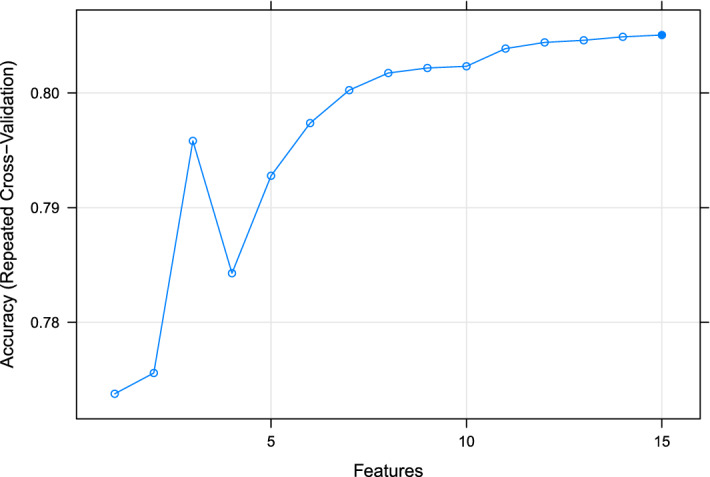
Illustration of the recursive feature elimination results incorporating 1 to 15 features. A random forest method was used to predict the udder health status of dairy cows according to somatic cell count below or above 200,000 cells/mL. The number of features included within the model is reported on the x-axis and the accuracy of the model from tenfold cross validation repeated 100 times is on the y-axis.

Machine learning algorithms can run more efficiently if a reduction of independent features is first performed, as some of these could be uninformative. In our study, recursive feature selection applied before training the algorithms revealed that the most parsimonious model with best performance (higher accuracy) was the one that included all investigated features (Fig. 1). Therefore, all 15 features were relevant in predicting the outcome and were considered for subsequent analysis.

### Method comparison and evaluation of performance in predicting udder health status on testing set

**Figure 2:**
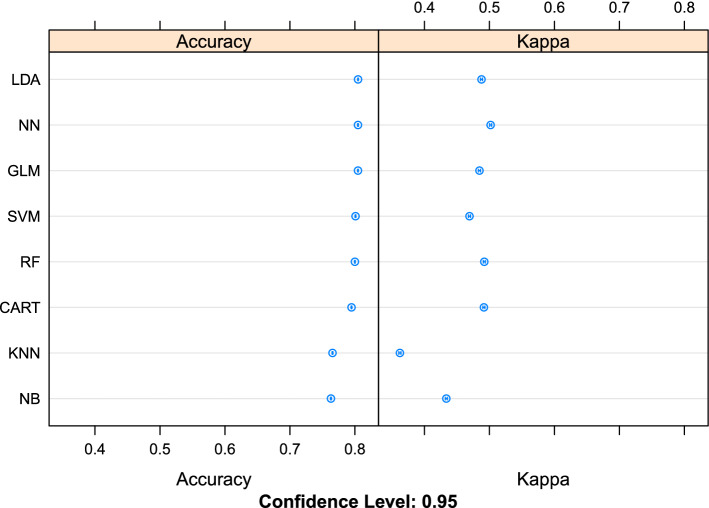
Comparison of the performance (accuracy and kappa value) of eight machine learning methods [Linear Discriminant Analysis (LDA), Neural Network (NN), Generalized Linear Model (GLM) with logit link function, Support Vector Machines (SVM), Random Forest (RF), Classification and Regression Trees (CART), k-Nearest Neighbors (kNN) and Naïve Bayes (NB)], run on the testing set, in prediction of udder health status of dairy cows according to somatic cell count below or above 200,000 cells/mL from cow’s and milk data recorded at the previous test-day record.

**Figure 3:**
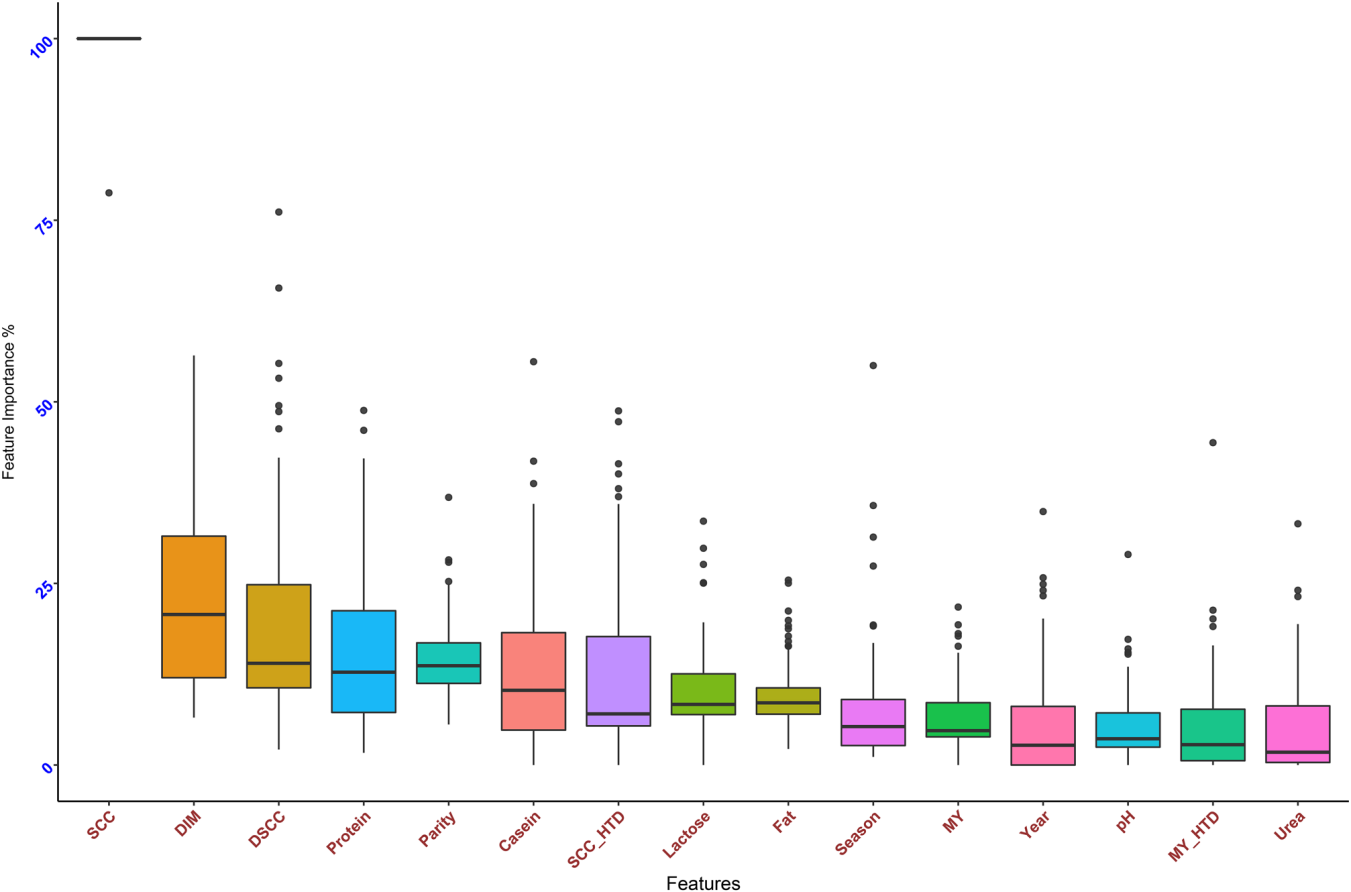
Plot of the feature importance across 100 replicates showing the features’ ranking for the prediction of udder health status of dairy cows according to somatic cell count below or above 200,000 cells/mL. Evaluated features, using neural network as predictive methods, are: log-transformed somatic cell count (log_10_SCC), stage of lactation (DIM), differential somatic cell count (DSCC), protein, parity order, casein, log_10_SCC of cows sampled in the same herd and day (log_10_SCC_HTD), lactose, fat, season of sampling, milk yield (MY), year of sampling, pH, MY of cows sampled in the same herd and day (MY_HTD) and urea.

Evaluation of performance of each ML method in predicting udder health status on testing set was based on accuracy and kappa values. Accuracies ranged from 76.3% (NB) to 80.5% (LDA, NN and GLM), whereas kappa values ranged from 36.2% in kNN to 50.2% in NN (Fig. 2). According to considered metrics, NN was the best method in predicting udder health classes (SCC ≤ 200,000 cells/mL or SCC > 200,000 cells/mL). Feature importance using NN on testing set for predicting udder health status suggested that log_10_SCC was the most important feature (as expected), followed by stage of lactation, DSCC, protein and parity (Fig. 3). Urea was the least informative feature.

### Method comparison and evaluation of performance in predicting udder health status on validation set

Method performance metrics on external validation set are summarized in Table 1. The LDA and RF methods showed the highest accuracy (79.7%) in predicting udder health classes, followed by GLM and NN (79.6%). The lowest accuracy (75.3%) was observed for the NB method. Sensitivity ranged from 61.6% (CART) to 38.1% (kNN). Among the other methods, sensitivity lower than 50% was reported only for SVM (47.9%). High specificity was observed for SVM (91.9%), GLM (91.3%) and LDA (90.9%). The best precision, or positive predictive value (PPV), was obtained for SVM (70.6%), GLM (70.6%) and LDA (70.3%), and the highest negative predictive value (NPV) was observed for CART (84.6%), NN (83.4%) and NB (83.1%). The CART method had also the greatest kappa value (49%) and F1 score (63.4%). The greatest values of Matthew's Correlation Coefficient (MCC) were observed for CART (0.490), NN (0.482) and RF (0.482).

**Table 1:** Methods performance metrics [accuracy and 95% confidence interval (CI), sensitivity (Se), specificity (Sp), positive predictive value (PPV), negative predictive value (NPV), kappa, F1 score and Matthew's Correlation Coefficient (MCC)] on external validation set. Prediction models were developed within different methods: Linear Discriminant Analysis (LDA), Generalized Linear Model (GLM) with logit link function, Naïve Bayes (NB), Classification and Regression Trees (CART), k-Nearest Neighbors (kNN), Support Vector Machines (SVM), Random Forest (RF) and Neural Network (NN).

Method	Accuracy	95% CI	Se	Sp	PPV	NPV	kappa	F1 score	MCC
LDA	0.797	0.784–0.810	0.524	0.909	0.703	0.823	0.468	0.600	0.478
GLM	0.796	0.783–0.809	0.511	0.913	0.706	0.820	0.461	0.593	0.472
NB	0.753	0.739–0.767	0.593	0.819	0.573	0.831	0.407	0.583	0.408
CART	0.793	0.780–0.806	0.616	0.865	0.652	0.846	0.490	0.634	0.490
kNN	0.754	0.740–0.768	0.381	0.907	0.626	0.781	0.325	0.474	0.342
SVM	0.791	0.777–0.804	0.479	0.919	0.706	0.812	0.439	0.571	0.454
RF	0.797	0.783–0.810	0.553	0.897	0.687	0.830	0.477	0.613	0.482
NN	0.796	0.782–0.808	0.567	0.889	0.677	0.834	0.479	0.617	0.482

**Figure 4:**
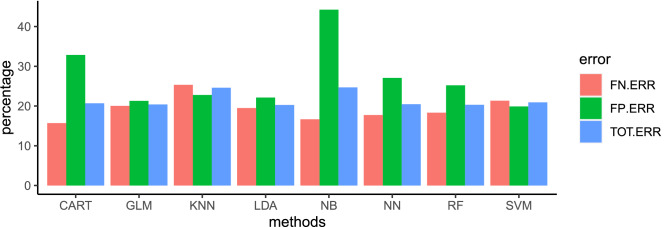
Comparison of the performance [false negative error (FN.ERR), false positive error (FP.ERR) and total error (TOT.ERR)] of eight machine learning methods [Classification and Regression Trees (CART), Generalized Linear Model (GLM) with logit link function, k-Nearest Neighbors (kNN), Linear Discriminant Analysis (LDA), Naïve Bayes (NB), Neural Network (NN), Random Forest (RF) and Support Vector Machines (SVM)] run on the validation set, in prediction of udder health status of dairy cows according to somatic cell count below or above 200,000 cells/mL from cow’s and milk data recorded at the previous test-day record.

Classification errors (false positive, false negative and total), based on method accuracy, are depicted in Fig. 4. The false negative error was higher for kNN (25.2%) than other methods, whereas the lowest error was reported for CART (15.7%). The highest false positive error was estimated for NB (44.3%) and CART (32.9%), and the lowest for SVM (19.9%). Considering the total error, the worst performances were reported for NB (24.6%) and kNN (24.5%), whereas the lowest error was observed for LDA (20.2%), GLM (20.3%), RF (20.3%) and NN (20.4%).

**Figure 5:**
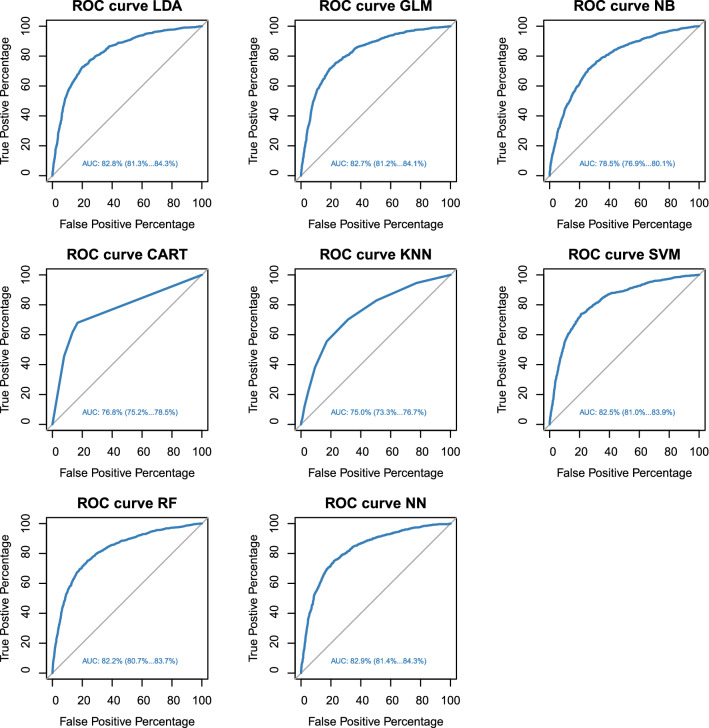
Comparing Receiver Operating Characteristic (ROC) curves of eight machine learning methods [Linear Discriminant Analysis (LDA), Generalized Linear Model (GLM) with logit link function, Naïve Bayes (NB), Classification and Regression Trees (CART), k-Nearest Neighbors (kNN), Support Vector Machines (SVM), Random Forest (RF) and Neural Network (NN)] run on the validation set, in prediction of udder health status of dairy cows according to somatic cell count below or above 200,000 cells/mL from cow’s and milk data recorded at the previous test-day record. In each plot, area under the curve (AUC) and 95% confidence interval were reported.

The Neural network showed the highest area under the curve (AUC, 82.9%), followed by LDA (82.8%) and GLM (82.7%); these methods had the best receiver operating characteristic (ROC) curves (Fig. 5). A lower AUC with a value of 75.0% was reported for kNN.

## Discussion

Machine learning algorithms offer new approaches for the analysis of large amount of data collected in dairy farming thanks to the use of herd management systems^25^ and milk testing performed in the frame of national recording procedures. Machine learning methods are indeed a promising tool to improve decision support systems for farmers and have already been applied in different areas of dairy research such as behaviour, feeding, management, physiology and reproduction^25^. Such advanced analyses allow to predict outcomes of economic relevance. In addition, the advantage of ML approaches over traditional statistical methods is the data-driven evaluation of the relationship between features and outcome, without bias introduced by the researcher’s hypothesis (e.g., assumption of linearity)^26^. The possibility to predict SCC increase using existing data (e.g., herd and cow’s data, as well as milk production and composition) provides an effective tool to improve the prevention of mastitis. In the present study we predicted the udder health status of cows using information collected at the previous TD across different herds, applying a repeated tenfold stratified cross-validation (CV), which is a robust method to prevent model overfitting^27^. In addition, similar performance of investigated methods on both testing and validation sets indicated the absence of overfitting. These results suggested the possibility to apply findings of this study also to other dairy herds^22^.

Performance of ML methods on testing and validation sets was evaluated by means of different metrics, such as accuracy, true and false positive rates, precision, F-score, and AUC value. Although it has become a common practice to provide several evaluation metrics, different measures assume a different use case^28^. Accuracy, which represents the number of correct predictions made by the model over the total number of predictions, is one of the most used metrics, however it is not sufficient alone to evaluate model performance. Indeed, depending on the specific case, the cost of false positive and of false negative might not be the same. Thus, model evaluation should be based also on other metrics, e.g. recall or sensitivity (if we want to minimize false negatives) and precision or PPV (if we want to minimize false positives). To get the model with best performance optimizing both false positive and false negative proportion, F1 score should be considered, as it represents the harmonic mean of both precision and recall. The AUC is another widely used metric for evaluation of classification problems, and it has the big advantage of being independent from outcome rate, as well as MCC. According to several of these metrics, NN, RF and linear models (LDA and GLM) were the methods with the best performance in predicting udder health status both in the testing and in the validation set. Among all, KNN had the worst performance, possibly due to its susceptibility to the presence of noise^29^. In detail, the best four methods had the lowest total error percentage for predictions on validation set, with NN and RF having among the lowest false negative error, and linear methods among the lowest false positive error. The CART method showed a good total error percentage comparable to that of NN, RF, LDA and GLM; however, together with NB, it had the lowest false negative, but the greatest false positive error. In addition, CART showed also a relatively poor performance based on AUC value. Lowering false negatives (i.e., erroneously classifying cows as healthy on the subsequent TD) balancing also the false positives would represent the best approach for our specific case. Thus, NN, RF and the linear methods seem to be the best methods in predicting udder health status. Nevertheless, although some methods had better performance than others, prediction accuracies of all methods were above 75%, meaning that on a herd with 100 cows we can correctly predict at least 75 cows. The good prediction ability of different NN methods applied on milk data has been previously reported in the literature^30,31^. Using instead a RF algorithm, Hyde et al.^22^ correctly replicated mastitis diagnosis at herd level with high degree of accuracy when compared with a veterinary clinician, highlighting the potential for such algorithms to reproduce the complex clinical diagnosis.

In our study, we applied a classification approach to predict subclinical mastitis. Predictions of udder health related outcomes based on SCC cut-offs were reported also by other authors^30–32^. In a previous study^19^, ML methods were applied to predict mastitis (SCC ≥ 250,000 cells/mL) using milking traits recorded at the same milking time by automatic systems. In addition, overall weights of milking traits in predicting subclinical mastitis based on investigated algorithms revealed that electrical conductivity and lactose percentage were the features with greater importance. In our study, the most important trait for prediction of udder health status based on NN was log_10_SCC, followed by stage of lactation, DSCC, protein and parity. Somatic cell count measured at the previous TD was expected to be the most important feature. Stage of lactation and parity are well-known factors affecting SCC variation^33^, whereas changes in protein profile of milk with high SCC due to proteolysis of casein, decreased synthesis of whey proteins, and leakage through the blood–milk barrier are well documented in the literature^34^. A positive correlation between SCC and DSCC was reported in a previous study^35^, as well as the combined use of DSCC and SCC to better screen for udder health status^14^.

Predictions obtained using different ML methods on data collected at different time points were reported by Anglart et al.^24^, who predicted cow composite SCC by using quarter and cow milk data regularly recorded in cows milked in an automatic milking system in a 8-week trial. The authors evaluated three ML methods (generalized additive model, RF, and multilayer perceptron), developing models with different variables setups, and found generalized additive model and multilayer perceptron to be promising for udder health prediction. Also, Ankinakatte et al.^36^ reported generalized additive model to be a good predictive method to detect oncoming clinical mastitis with automated recorded data, together with NN. Results of the present study further highlight the good predictive ability of linear and NN methods. Nevertheless, an interesting aspect has arisen from our analyses, by performing some tests using non-transformed and log-transformed SCC traits (SCC and SCC_HTD). Our findings suggested that performances of most of the analyzed methods were not affected by variable transformation (supporting the hypothesis that no transformation should be required in ML analyses), whereas performances of linear methods (GLM and LDA) improved using log-transformed SCC traits. Indeed, the accuracy increased by about 2%, the AUC by 4%, the MCC by 8% and the F1 score by 10%. For this reason, we reported results obtained with log-transformed SCC traits and highlighted the data transformation effect on linear ML methods performances. This aspect should be considered when choosing the ML methods to be applied to a specific analysis.

Anglart et al.^24^ also suggested to include information on cows’ previous composite SCC in model training to lower the prediction error. Although we recognize that predictions between monthly TD sampling would represent an asset for mastitis monitoring, such application is feasible only if automatic milking systems are used by the farmers, which is not the case of the current situation in Italy.

The low to moderate sensitivity (38–62%) and relatively high specificity (> 82%) of all ML methods evaluated in the present study suggested their high power to correctly identify healthy cows (still a valuable result in order to narrow the group of animals that could possibly develop the disease) and the scarce ability to distinguish subclinical mastitis. Considering NN, 290 false positive out of 1071 true positive records and 464 false negative out of 2616 true negative records were obtained. However, false positives were classified as mastitic although healthy at the subsequent TD with an average probability of 0.67 (in a 0–1 scale, where values above 0.50 define the prediction as “mastitic” and values below 0.50 as “healthy”; data not shown). Thus, probability estimates rather than only a binary outcome should be taken into account to define the uncertainty of a prediction. In detail, a probability of 0.51 of being mastitic is less reliable than a probability of 0.99 and a farmer should be aware of such differences within each class (healthy or mastitic). In addition, 254 out of 290 records had SCC greater than 200,000 cells/mL in the previous TD, driving the prediction to define the animals as mastitic. These cows possibly spontaneously recovered from a mastitic event. Nevertheless, high SCC level in the previous TD suggests alone that special attention should be placed to these animals, independently from ML future predictions. Similarly, for NN method, 384 out of 464 records that were classified as healthy but should have been considered mastitic had SCC at the previous TD lower than 200,000 cells/mL. These cows could have undergone a mastitic event in the time between two TD; thus, reduction in the time period between TD recording would likely improve prediction accuracy. Nevertheless, 149 out of 384 false negatives had, at the previous TD, SCC below 200,000 cells/mL but DSCC above 70%, a threshold that is currently used to distinguish the udder health status. Indeed, cows with low SCC and high DSCC are considered as susceptible to mastitis^14^. Therefore, our results provide further evidence on the important information provided by DSCC, in combination with SCC.

Despite the large number of published studies on ML methods applied to animal science, a reliable practical implementation of most tested algorithms for management decision has not occurred yet. This may be due to the availability of poor training data^25^. To improve prediction accuracies, data retrieved from many different herds and recorded for longer time periods should be considered, and large integrated high-quality datasets have to be created. Another possibility would be the development of more herd-specific algorithms, focusing on the specific needs of individual farmers.

Our results underlined the difficulty in defining the best methods to be used in ML analysis, as some methods might be affected by data structure and distribution. Nevertheless, findings of this study represent a valuable prelude to develop ML models that will provide to the farmers a prediction of whether their cows will possibly have high SCC level at the subsequent TD using already available information.

## Methods

### Data collection and editing

Cows in the current study belonged to commercial herds and were not subjected to any invasive procedures. Milk samples were previously collected during routine milk recording procedures by the personnel of “the Breeders Association of Veneto Region” (Padova, Italy) and thus data for the study, collected from January 2018 to January 2020, were retrieved from this Association. The dataset included information on herd, cows (ID, breed, stage of lactation and parity), date of sample collection, daily milk production (kg/d), milk composition [fat, protein, casein and lactose percentages, pH and urea (mg/100 mL)] determined through Milkoscan FT6000 (Foss, Hillerød, Denmark), SCC (cells/mL) and DSCC (%) determined using the Fossomatic 7 DC (Foss, Hillerød, Denmark). At the time of sampling, the laboratory of the Breeders Association of Veneto Region was equipped with 3 infrared instruments to determine somatic cells, 2 Fossomatic 6 (Foss, Hillerød, Denmark), which provided only SCC, and 1 Fossomatic 7 DC, which provided information on SCC and DSCC. Thus, approximately one third of all the milk samples collected in Veneto Region in the framework of the routine milk testing procedures and processed by the laboratory could be randomly analyzed using the Fossomatic 7 DC. Only milk samples that were analyzed using the Fossomatic 7 DC, that included the new DSCC trait, were used for subsequent statistical analysis, whereas the other records were excluded from the dataset. In addition, the original dataset was edited to select Holstein Friesian cows between 5 and 480 days in milk (DIM) and with a minimum of 2 TD records within lactation; other breeds were excluded from the analysis due to few observations available. Among milk traits, outliers beyond 4 standard deviations, possibly resulting from errors in milk sample collection, measurement or data entry procedures, were excluded from the subsequent statistical analysis. Furthermore, to avoid data fragmentation over time, only lactations with an interval between 2 consecutive TD < 6 weeks were considered, i.e. if more than 42 days occurred between two consecutive TD, such data were not considered for subsequent analysis. Records with missing values were discarded. Contemporary groups were defined as cows sampled in the same herd and day (herd-test-date, HTD). Average milk production (MY_HTD) and SCC (SCC_HTD) of contemporary groups were also determined. According to Valletta et al.^37^, there is a substantial difference between making inferences and predictions. In detail, statistical models focused on inference start with an assumption about data distribution (i.e., the normality assumption of the distribution of the dependent variable and the residuals). Machine learning models focus on predictions and do not assume a functional distribution of the data. For this reason, we initially decided to run the analysis with no data transformation. However, we also log-transformed SCC-related traits, which do not have normal distribution. Following these preliminary tests, the two SCC-related traits (SCC and SCC_HTD) were log-transformed to achieve normality, whereas no transformation was required for DSCC. A total of 15 features within each TD record were considered: parity (1, 2, 3 and ≥ 4), stage of lactation (11 classes of 30 d each and the last being an open class > 305 DIM), year of sampling (2018 and 2019), season of sampling (winter: December, January, February; spring: March, April, May; summer: June, July, August; autumn: September, October, November), milk yield (MY), fat, protein, casein, lactose, pH, urea, log_10_SCC, DSCC, MY_HTD and log_10_SCC_HTD. For each TD record *n*, udder health status at TD *n* + 1 (Outcome) was classified using a predefined SCC threshold of 200,000 cells/mL^38^; in detail, health status was coded as 0 for SCC ≤ 200,000 cells/mL and 1 for SCC > 200,000 cells/mL. After editing, the final dataset included 18,442 records of 14,064 cows in 791 herds. Selected farms had an average herd size of 135 milking cows, ranging from 4 to 1,546. Most of them presented traditional milking parlor, whereas only few herds were equipped with automatic milking systems. These 791 farms had an average cow’s milk production of 30.7 kg/day, and the average fat and protein content was 4.03% and 3.45%, respectively. The much lower mean herd size of the subset analyzed (~ 18 cows) was due to the restrictive data editing previously described. Each record included information of 2 subsequent TD: outcome at TD *n* + *1* and independent features of TD *n*. The prevalence of subclinical mastitis (SCC > 200,000 cells/mL) was 29.0%.

### Data processing

Within eight different ML methods (LDA, GLM, NB, CART, kNN, SVM, RF and NN), prediction models were developed to identify the best udder health prediction method based on cow and milk data of the previous TD. The dataset of 18,442 records was randomly split into two subsets: 80% of the data (14,755 records) were used to train and test the models, whereas the remaining 20% of the data were excluded from model construction and held back as an external validation set. Recursive feature selection using a tenfold CV repeated 100 times with RF method was applied to automatically select a subset of the most predictive features to identify the most parsimonious model with best performance.

To train and test the relationship between outcome (0/1 based on SCC levels at TD *n* + 1) and features (cow and milk data of TD *n*), a stratified tenfold CV repeated 100 times was employed. Stratification allowed to take into account the imbalanced output classes and to preserve the percentage of samples for each target class^27^. The original train/test dataset (n = 14,755) was randomly divided into 10 subsets of equal size. Prediction models were trained on 9 of these subsets and the last subset was used as test set to evaluate methods performance in predicting the outcome. Each tenfold CV was repeated 100 time. A total of 1,000 iterations were performed and 100 mean accuracy value of the tenfold CV were then averaged to obtain the final accuracy of each method reported in the tables. Standardization of data (center and scale) was performed within CV. Tuning details of each model are reported in Supplementary file 1. Data analysis was performed using Caret v.6.0–86^39^ and Tidyverse v. 1.3.1^40^ packages of R software v.4.0.5 (https://cran.r-project.org/bin/windows/base/)^41^.

### Method comparison and evaluation of performance on validation set

Accuracy of each prediction model on testing set was used as first comparison metric among algorithms. The accuracy of a test represents the proportion of correct predictions (true positive and true negative) among all examined cases. Feature importance using the best prediction model (the one with the greatest accuracy) on testing set was assessed. Predictive skills (predicted outcome 0/1, as well as predicted probability from 0 to 100% of being classified as 0 or 1) of all investigated models on external validation set were then estimated. Results were analysed via a confusion matrix and several performance statistics [e.g., accuracy, sensitivity, specificity, positive predictive value (PPV) and negative predictive value (NPV)] on validation set were reported for each method.

False positive, false negative and total error rates across methods were also compared. Cohen’s Kappa value, which is a measure of the classifier’s performance as compared to its performance simply driven by chance, and F1 score, which is a weighted mean of sensitivity and PPV, were reported. Receiver operating characteristic curve analysis was performed using pROC package v. 1.17.0.1^42^ in R and the AUC value was used for methods performance comparison. In addition, as a measure of the classification’s quality, MCC for each method was calculated according to the following formula:

$${\text{MCC}} = \frac{{TP~ \times TN - FP~ \times FN}}{{\sqrt {\left( {{\text{TP}} + {\text{FP}}} \right) \times \left( {TP + FN} \right) \times \left( {TN + FP} \right) \times \left( {TN + FN} \right)} }}$$

where TP is true positive, TN is true negative, FP is false positive and FN is false negative.

## Supplementary Information

Supplementary Information.

## Data Availability

The dataset analysed in the current study is available from the corresponding author on reasonable request.
