# Supplementary material for: Comparison of machine learning methods to predict udder health status based on somatic cell counts in dairy cows
Source: Sci Rep. 2021 Jul 1;11:13642. doi: 10.1038/s41598-021-93056-4 (PMC8249463; doi:10.1038/s41598-021-93056-4)
Supplement: Supplementary file 1 — Supplementary Information. [file 41598_2021_93056_MOESM1_ESM.pdf]

Comparison of machine learning methods to predict udder health status based on somatic cell counts in dairy cows

Tania Bobbo[1,*], Stefano Biffani[2], Cristian Taccioli[3], Mauro Penasa[1], and Martino Cassandro[1]

[1]Department of Agronomy, Food, Natural resources, Animals and Environment (DAFNAE), University of Padova, Viale dell'Università 16, 35020 Legnaro (PD), Italy
[2]Istituto di Biologia e Biotecnologia Agraria, Consiglio Nazionale delle Ricerche, Via Edoardo Bassini 15, 20133 Milano, Italy
[3]Department of Animal Medicine, Production and Health (MAPS), University of Padova, Viale dell'Università 16, 35020 Legnaro (PD), Italy

Supplementary Information file. Parameters set for some of the algorithms used in this study.

Naïve Bayes (NB)
Resampling results across tuning parameters:

usekernel	Accuracy	Kappa
FALSE	0.7631081	0.43359359
TRUE	0.7267707	0.09699593

Tuning parameter 'fL' was held constant at a value of 0.
Tuning parameter 'adjust' was held constant at a value of 1.
Accuracy was used to select the optimal model using the largest value.
The final values used for the model were fL = 0, usekernel = FALSE and adjust = 1.

Classification and Regression Trees (CART)
Resampling results across tuning parameters:

cp	Accuracy	Kappa
0.01212687	0.7947766	0.4914436
0.01282649	0.7929237	0.4954891
0.28568097	0.7473059	0.2495228

Accuracy was used to select the optimal model using the largest value.
The final value used for the model was cp = 0.01212687.

k-Nearest Neighbors (kNN)
Resampling results across tuning parameters:

k	Accuracy	Kappa
5	0.7552525	0.3538142
7	0.7606182	0.3579216
9	0.7655360	0.3621878

Accuracy was used to select the optimal model using the largest value.
The final value used for the model was k = 9.

Support Vector Machines (SVM)
Resampling results across tuning parameters:

C	Accuracy	Kappa
0.25	0.8009516	0.4692618
0.50	0.8001465	0.4637676
1.00	0.7984143	0.4581571

Tuning parameter 'sigma' was held constant at a value of 0.02104279.
Accuracy was used to select the optimal model using the largest value.
The final values used for the model were sigma = 0.02104279 and C = 0.25.

Random Forest (RF)
Resampling results across tuning parameters:

mtry	Accuracy	Kappa
2	0.7953448	0.4472602
15	0.7999865	0.4920187
28	0.7973034	0.4853928

Accuracy was used to select the optimal model using the largest value.
The final value used for the model was mtry = 15.
The “rf" method in the Caret package sets the default number of trees at 500.

Neural Network (NN)
Resampling results across tuning parameters:

size	decay	Accuracy	Kappa
1	0e+00	0.8033859	0.5000201
1	1e-04	0.8036190	0.5007532
1	1e-01	0.8044614	0.5028761
3	0e+00	0.8033283	0.4981121
3	1e-04	0.8033649	0.4987079
3	1e-01	0.8047149	0.5017679
5	0e+00	0.8014789	0.4946348
5	1e-04	0.8012905	0.4940170
5	1e-01	0.8027761	0.4976747

Accuracy was used to select the optimal model using the largest value.
The final values used for the model were size = 3 and decay = 0.1.
